# Targeting Topoisomerase I and DNA with LCS1269 Drives Glioblastoma Cell Death Despite ATM/Chk1/BRCA1/RAD51 Signaling Pathway Activation

**DOI:** 10.3390/ijms26136014

**Published:** 2025-06-23

**Authors:** Nikolay Kalitin, Ekaterina Savchenko, Nadezhda Samoylenkova, Natalia Koroleva, Anna Lushnikova, Aida Karamysheva, Galina Pavlova

**Affiliations:** 1 Laboratory of Tumor Cell Genetics, N.N. Blokhin National Medical Research Center of Oncology, 115522 Moscow, Russia; aikaram@yandex.ru; 2 Laboratory of Molecular and Cellular Neurogenetics, N.N. Burdenko National Medical Research Center of Neurosurgery, 125047 Moscow, Russia; savhenko61@mail.ru (E.S.); samoylenkova.n@gmail.com (N.S.); lkorochkin@mail.ru (G.P.); 3 Laboratory of Oncogenomics, N.N. Blokhin National Medical Research Center of Oncology, 115522 Moscow, Russia; nat.korole@yandex.ru (N.K.); lan21@yandex.ru (A.L.); 4 Laboratory of Neurogenetics and Developmental Genetics, Institute of Higher Nervous Activity and Neurophysiology of RAS, 117485 Moscow, Russia

**Keywords:** LCS1269, Topoisomerase I, DNA damage, ATM, Chk1, BRCA1, glioblastoma

## Abstract

Glioblastoma (GBM) is the most aggressive primary brain tumor in adults. The success of modern multimodal standards approved in anti-glioblastoma therapy remains limited. Consequently, new therapeutics are urgently needed. In this study, utilizing ex vivo, in silico, and in vitro approaches, we investigated the LCS1269 effects on two potential targets, DNA and Top I. We also elucidated the influence of LCS1269 on signaling pathways and GBM cell viability. Based on our docking data and competition studies results, we demonstrated that LCS1269 may bind to DNA, demonstrating selectivity toward AT-rich regions. We also showed that LCS1269 could dock both Top I/DNA binary complex and Top I active sites. LCS1269 caused Top I dysfunction and downregulated the expression of Top I. Moreover, the LCS1269 treatment of GBM cells facilitated DNA damage and the activation of the ATM/Chk1/BRCA1/Rad51 pathway. Meanwhile, DNA damage response induction and ATM/Chk1/BRCA1/Rad51 pathway activation were insufficient to prevent GBM cell death triggered by LCS1269 treatment. Our work shows that DNA and Top I are promising molecular targets of LCS1269, thus providing insight on several novel mechanisms of its anti-tumor activity. Nonetheless, we did not perform a biophysical validation of the LCS1269–DNA interaction, which is a limitation of our study.

## 1. Introduction

Glioblastoma multiforme (GBM) is one of the most aggressive and intractable malignancies of the central nervous system (CNS), with the five-year survival rate being just around 7% [1]. Even though GBM rarely forms extracranial metastases, intratumor heterogeneity, a highly infiltrative nature, reliable DNA repair systems of GBM cells, the exceptional self-renewing capability of glioblastoma stem cells, and blood–brain barrier protection account for resistance toward modern chemotherapeutics [2,3,4,5]. Consequently, new treatment opportunities circumnavigating these challenges are urgently needed.

Despite the development of novel, state-of-the-art anti-tumor therapy approaches over the last few years, therapeutic methods aiming to destroy cancer cells remain a valuable therapeutic approach. DNA continues to be an essential target for many novel antitumor DNA binding compounds that target DNA either to affect DNA-associated processes or to trigger DNA damage [6].

Top I is a ubiquitously expressed enzyme which relaxes supercoiled DNA during various cellular processes such as replication, transcription, and recombination [7]. It has been shown that *Top I* expression was much higher in glioblastoma cell lines as compared to the non-malignant cells [8], thus suggesting that its inhibition could provide an opportunity to reduce the relaxation of supercoiled DNA of GBM cells. Indeed, Top I inhibitor, irinotecan, was used in clinical trials as a second-line drug for GBM treatment. Irinotecan demonstrated an overall objective response rate of 14 to 15%. However, stabilization of the disease has been achieved in 14 to 55% [9,10]. Another Top I inhibitor, topotecan, was evaluated for treatment in refractory and relapsed GBM patients [11]. In addition, several clinical studies have demonstrated that topotecan sensitized human GBM cells in both adults and children to radiotherapy [12,13,14,15]. Thus, new therapeutics designed to modulate Top I activity might be a key approach for GBM treatment considering the high proliferative activity of GBM cells [16].

LCS1269 is an indolo [2,3-*a*] pyrrolo [3,4-*c*] carbazole derivative tethered via one N-glycosidic bond of an indolopyrrolocarbazole skeleton with xylose fragment and linked through the amino (NH) group with a nitrogen atom of the pyrrole cycle and picolinic acid moiety (Supplementary Figures S1 and S2). LCS1269 has shown promising in vitro and in vivo potency toward various cancer cell lines, including breast, colon, and lung cells, as well as murine and human tumor models [17]. In addition, LCS1269 was found to overcome multidrug resistance and to inhibit vasculogenic mimicry in melanoma cells [18]. It was also demonstrated that LCS1269 significantly reduced the tumor volume mass of B-16 transplantable melanoma [19]. Moreover, we have previously identified LCS1269 as a potential anti-glioblastoma agent, probably interfering with cell cycle regulation through both direct CDK1 inhibition and indirect modulation of CDK1 activity through Wee1/Myt1 and FOXM1/Plk1 signaling pathways [20]. However, several questions remained unanswered during our previous study, and therefore we believed it was important to address them.

Firstly, we aimed to explore the mechanism by which LCS1269 could interact with DNA and, consequently, damage it in GBM cell lines. Secondly, we sought to investigate the effect of LCS1269 treatment on Top I activity and its expression. In addition, we examined the influence of LCS1269 on DNA damage response induction, reparation, and further fate of GBM cells.

## 2. Results

### 2.1. LCS1269 Interacts with DNA Through Minor Groove Binding Rather than in an Intercalative Manner

**Figure 1:**
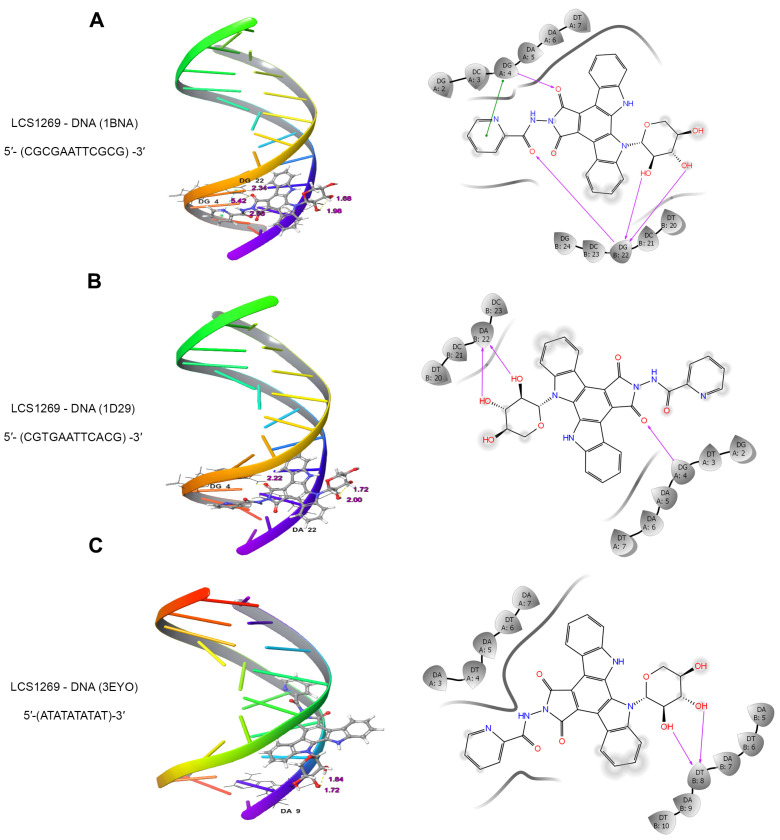
LCS1269 targets DNA and preferentially binds to AT-rich sequences. (**A**–**C**) The 3D binding models (**left**) and 2D diagrams (**right**) showing the simulated interactions between LCS1269 and nucleotide residues of DNA duplexes 5′-CGCGAATTCGCG-3′ (PDB ID: 1BNA), 5′-CGTGAATTCACG-3′ (PDB ID: 1D29), and 5′-ATATATATAT-3′ (PDB ID: 3EYO). Purple arrows show hydrogen bonds while green lines illustrate π–π stacking interactions.

Using a fluorescent intercalator displacement assay [21], it has previously been shown that LCS1269 could bind to DNA (see Supplementary Note S1 for more details). However, our colleagues were not able to unambiguously define the mechanism of interaction between LCS1269 and DNA (Supplementary Note S1 and [22]) utilizing a biophysical approach such as circular dichroism in cholesteric liquid crystals [23]. Therefore, in the current study, our aim was to identify the mechanism underlying the interaction of LCS1269 with DNA. To this end, we firstly docked LCS1269 to three double-stranded DNA fragments by molecular modeling (Figure 1A–C).

Computational analysis of the interactions between LCS1269 and dodecamer duplex (PDB ID: 1BNA) sequence d(CGCGAATTCGCG)_2_ in a minor groove of DNA B-form proposed π–π stacking interaction between the benzene ring of picolinic acid and G4 combined with an acceptor H-bond between oxygen in the pyrrole ring and the same guanine. The G22 formed two donor H-bonds with 2′-OH and 3′-OH groups in carbohydrate moiety and an acceptor H-bond with an oxygen atom (Figure 1A).

Another dodecamer duplex (PDB ID: 1D29) includes nucleotide substitutions C–>T and G–>A in the sequence 3′- CGCGAATTCGCG-5′, resulting in the sequence 3′-(CGTGAATTCACG)-5′ [24]. The 1D29 interaction with LCS 1269 simulated one acceptor H-bond between G4 and oxygen in the pyrrole ring and two H-bonds between A22 and 2′-OH and 3′-OH groups in the sugar moiety of LCS1269 (Figure 1B).

Finally, the coiled-coil structure formed by the complex of DNA duplex d(ATATATATAT)_2_ with pentamidine was described [25]. This duplex (PDB ID: 3EYO) was found to have a mixed structure containing Watson–Crick and Hoogsteen base pairs. The drug pentamidine stabilizes the coiled coil through the formation of cross-links between two DNA duplexes. The central part of the drug was found in the minor groove, as expected, whereas the charged terminal amidine groups protruded and interacted with phosphates from DNA helix chains. The formation of cross-links may be related to the biological effects of pentamidine, which is used as an antibacterial and antiviral agent. Obviously, AT-rich DNA sequences are highly abundant in eukaryotic genomes. However, very few data are available on DNA sequences which contain AT base pairs only. Docking analysis predicted that LCS1269 also interacted with this duplex by means of two donor H-bonds between T8 and 2′-OH and 3′-OH groups in sugar moiety and parallel orientation between the DNA duplex (Figure 1C). This configuration score was rather high (>–7), indicating that LCS1269 might preferentially target AT-rich regions in genomic DNA (Supplementary Table S1).

**Figure 2:**
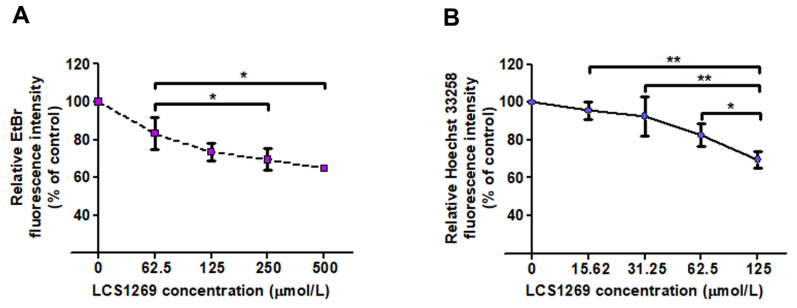
LCS1269 interacts with DNA as both an intercalative agent and minor groove binder. (**A**) DNA binding affinity determined by the competition of LCS1269 with intercalator ethidium bromide utilizing a spectrofluorometric technique. (**B**) DNA binding affinity assessed by the competition of LCS1269 with minor groove binder Hoechst 33,258 by spectrofluorometric technique. Data presented as mean ± SD. * *p* < 0.05 and ** *p* < 0.01.

To verify our docking studies, we further evaluated DNA-interacting properties of LCS1269 based on the replacement of the DNA intercalating agent ethidium bromide (EtBr) and the minor groove binder Hoechst 33258. The background fluorescence for EtBr and Hoechst 33,258 solutions in the presence of LCS1269 was 140.79 ± 4.73 and 77.27 ± 5.98, respectively, whereas the fluorescence of EtBr and Hoechst 33,258 solutions without LCS1269 was 134.63 ± 19.49 and 75.13 ± 8.21, respectively. The baseline fluorescence intensities of the solutions containing either double-stranded *Salmon sperm* DNA (SS-DNA) with EtBr or SS-DNA with Hoechst 33,258 were 2030.54 ± 204.03 and 3514.06 ± 257.38, respectively. The dose-dependent incubation of LCS1269 with SS-DNA, EtBr, or Hoechst 33,258 was followed by spectrofluorometric analysis. The intensities of fluorescence emissions from EtBr and Hoechst 33,258 were significantly decreased with increasing concentrations of LCS1269, reaching about 60% and 70%, respectively (Figure 2A,B).

In summary, LCS1269 appeared to be more effective in Hoechst 33,258 competition assay and to have a higher affinity to the minor groove of DNA.

### 2.2. LCS1269 Inhibits Enzyme Activity and Alleviates Top I Protein Expression

**Figure 3:**
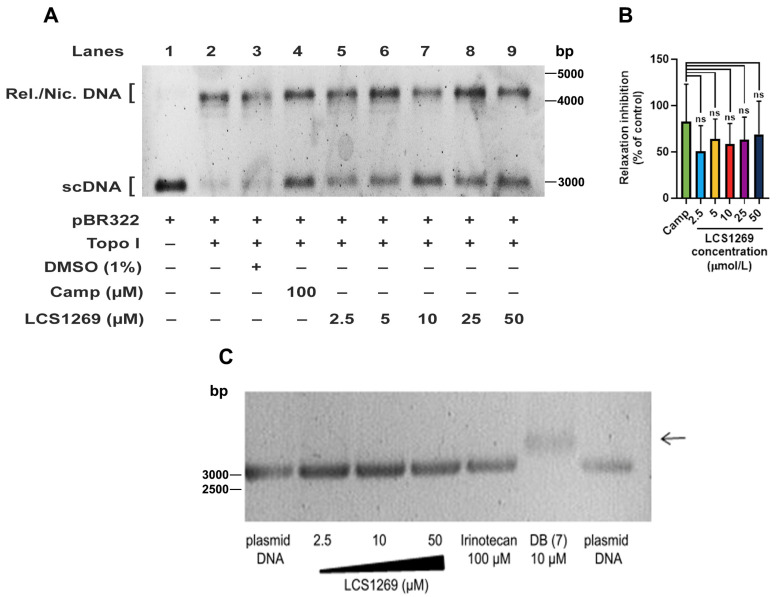
LCS1269 inhibits Top I enzyme activity. (**A**) Relaxation analysis of supercoiled pBR322 DNA. MCF7 whole cell extract was used as a source of endogenous Top I. Lane 1, supercoiled plasmid DNA (pBR322, scDNA, 100 ng). Lane 2, scDNA + MCF7 lysate. Lane 3, scDNA + MCF7 lysate sample incubated with 1% DMSO. Lane 4, 100 µM camptothecin (Camp) added to scDNA + MCF7 lysate sample (positive control). Lane 5–9, scDNA + MCF7 lysate samples incubated with increasing concentrations of LCS1269. Positions of supercoiled and relaxed/nicked DNA are indicated as scDNA and Rel./Nic. DNA, respectively; bp—base pairs. (**B**) Quantification of relaxation inhibition (%) in samples treated with increasing concentrations of LCS1269 or 100 µM camptothecin (Camp); ns—not significant. (**C**) Electrophoretic mobility pattern of untreated supercoiled pBR322 (plasmid DNA) and pBR322 DNA treated with indicated compounds. Black arrow delineates the migration behavior of plasmid DNA (and delay compared to other bands) treated with a strong DNA binder DB (7); bp—base pairs.

During transcription and genome duplication processes, Top I removes positive and negative supercoils of DNA by producing transient breaks to the DNA backbone followed by the re-ligation of the cleaved strands [26]. The stabilization of transient covalent Top I-DNA cleavage complexes by Top I inhibitors, such as camptothecin, results in DNA damage. We found that there was LCS1269 dose-dependent inhibition of Top I activity as determined by a relaxation analysis of supercoiled pBR322 DNA (Figure 3A,B). The inhibition reached 68.78 ± 35.88% after LCS1269 treatment at maximum concentration (50 µM). In comparison, the inhibition rate of the clinically approved Top I inhibitor, camptothecin (positive control), was 82.73 ± 40.27% (*p* > 0.05). No significant differences in relaxation inhibition between camptothecin and other LCS1269 concentrations (2.5, 5, 10, and 25 µM) were found either (Figure 3B).

Even at low concentrations of 2.5 µM and 5 µM, LCS1269 considerably inhibited the relaxation activity of Top I, although a complete inhibition was never achieved under these conditions (Figure 3A, lanes 5 and 6). Notably, Top I activity was evidently reduced after using higher concentrations of LCS1269 (10 µM, 25 µM, and 50 µM) (Figure 3A, lanes 7, 8, and 9).

To investigate whether LCS1269 binds to DNA and potentially influences Top I unwinding activity, we carried out a pBR322 DNA gel-migration assay in the presence of the same LCS1269 doses that were used in the relaxation assay earlier. As shown in Figure 3C, the incubation of plasmid DNA with increasing concentrations of LCS1269 did not slow the migration of pBR322 DNA. On the other hand, the strong DNA binder DB (7) slowed down the plasmid migration (black arrow). These results are consistent with our EtBr competition studies, in which we demonstrated that LCS1269 could significantly intercalate into DNA at a dose of 250 µmol/L and higher. Therefore, we concluded that LCS1269-induced Top I inhibition could be mediated by an influence on the Top I activity itself rather than by DNA intercalation.

**Figure 4:**
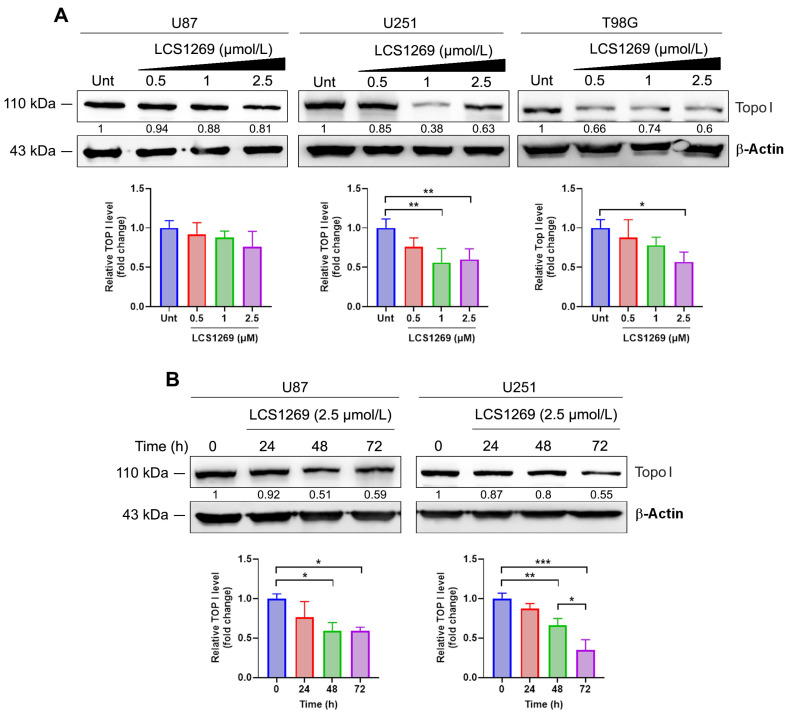
LCS1269 downregulates the Top I protein level in both a dose- and time-dependent manner. (**A**) Western blot analysis of Topoisomerase I protein levels in U87, U251, and T98G cells treated with LCS1269 in indicated concentrations for 24 h. Bar charts show the relative band density of Top I normalized to β-Actin. (**B**) Western blot analysis of Topoisomerase I protein levels in U87 and U251 cells after LCS1269 treatment for 24–72 h. Bar charts indicate the relative band density of Top I normalized to β-Actin, respectively. β-Actin was used as a loading control. Data presented as mean ± SD. * *p* < 0.05, ** *p* < 0.01, and *** *p* < 0.001.

LCS1269 caused the dose-dependent inhibition of Top I protein expression in all glioblastoma cells studied (Figure 4A). The inhibitory effect of LCS1269 was enhanced in U87 and U251 cells after 24–72 h of incubation (Figure 4B). Since the expression of the protein could be depleted by the inhibition of the appropriate mRNA expression and/or by proteasome pathway degradation of the protein itself, we examined the impact of LCS1269 on the proteasomal-dependent degradation of Top I. As illustrated in Supplementary Figure S3, LCS1269 treatment did not influence the proteasome pathway, suggesting that the downregulation of Top I expression might be proteasome-independent.

**Figure 5:**
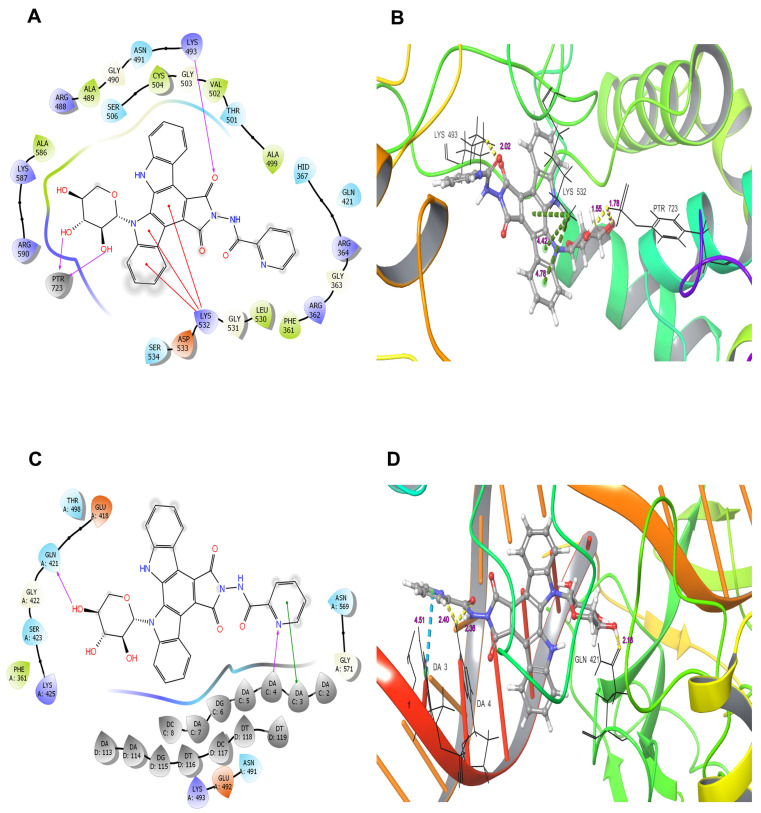
Proposed binding modes of LCS1269 with Top I (PDB ID: 1A31). (**A**) The 2D diagram showing hypothetical interactions formed by ligand LCS1269 within the active site of Top I without DNA (the purple arrows show hydrogen bonds while the red lines denote cation–π interactions). (**B**) The 3D cartoon model illustrating the LCS1269 binding mode with Top I enzyme only (the yellow dashed lines represent hydrogen bonds and green dashed lines mean cation–π interactions. (**C**) The 2D diagram predicting interactions formed between LCS1269 and the active site of Top I in complex with DNA (purple arrows show hydrogen bonds and green lines demonstrate π–π stacking interactions). (**D**) The 3D model of interactions simulated between LCS1269 and Top I/DNA complex (yellow dashed lines denote hydrogen bonds and blue dashed lines mark π–π stacking interactions).

We further investigated the ability of LCS1269 to interact with the Top I (1A31) active center alone using molecular docking. Two donor H-bonds of phosphorylated Tyr723 (PTR723) with 2′-OH and 3′-OH groups in the carbohydrate moiety and the acceptor H-bond between the oxygen of the pyrrole ring and Lys493 were predicted. In addition, three cation–π interactions between the pyrrole and benzene rings of the LCS1269 molecule and the positively charged Lys532 residue were also proposed (Figure 5A). The glide score value for the aforementioned simulation of LCS1269 interaction with Top I was –6.45. The lengths of the bonds in angstroms are shown in the 3D diagram (Figure 5B). Then, the computational analysis of LCS1269 hypothetical interactions between LCS1269 and the Top I/DNA complex was carried out. Our analysis revealed that 4′-OH group sugar moiety formed the donor H-bond with Gln421, whereas the nitrogen of picolinic acid formed the acceptor H-bond with A4 of the neighboring DNA fragment (Figure 5C). Additionally, a π–π stacking interaction was predicted between the benzene ring of picolinic acid and the A3 nucleotide. The glide score was –5.96. The lengths of these bonds in angstroms are shown in the 3D diagram (Figure 5D).

### 2.3. LCS1269 Triggers DNA Damage Propagation Followed by Cell Death in Glioblastoma Cells

**Figure 6:**
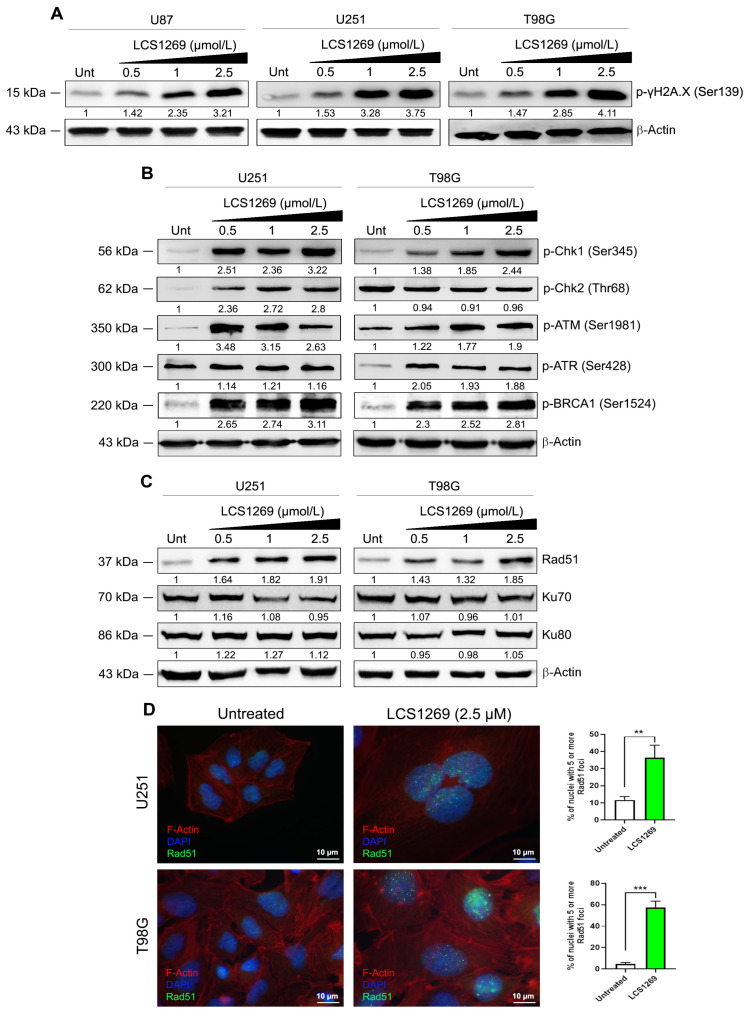
LCS1269-induced DNA damage followed by DNA damage response propagation through ATM/Chk1/BRCA1/Rad51 pathway. (**A**) Western blot analysis of phosphorylated histone p-γH2.AX (Ser139) protein levels in U87, U251, and T98G cells treated with LCS1269 in indicated concentrations for 24 h. (**B**) Western blot analysis of p-Chk1 (Ser345), p-Chk2 (Thr68), p-ATM (Ser1981), p-ATR (Ser428), and p-BRCA1 (Ser1524) protein levels in U251 and T98G cells treated with LCS1269 in indicated concentrations for 24 h. (**C**) Western blot analysis of Rad51, Ku70, and Ku80 protein levels in U251 and T98G cells treated with LCS1269 in indicated concentrations for 24 h. β-Actin served as a loading control. (**D**) Representative images of LCS1269 (2.5 µmol/L, 24 h)-induced Rad51 foci in U251 and T98G cells (magnification, ×100). Scale bar, 10 µm. Bar charts illustrate the percentage of Rad51 foci positive nuclei (with ≥5 Rad51 foci in nucleus) expressed from three independent experiments. Data presented as mean ± SD. ** *p* < 0.01 and *** *p* < 0.001.

DNA double-stranded breaks (DSBs) generated by DNA-damaging agents or ionizing radiation result in the rapid phosphorylation of histone H2AX at Ser 139 (p-γH2AX), which is known to be one of the first cellular responses to the introduction of double-strand breaks [27]. The increase in the phosphorylation level of H2AX (p-γH2AX (Ser 139)) due to the LCS1269 treatment in all tested glioblastoma cells was used as a readout and confirmation of DNA damage (Figure 6A).

The transducing kinases ATM (ataxia-telangiectasia mutated) and ATR (ATM and RAD3-related) are commonly activated by DNA damage sensors and play critical roles in early signal transmission through cell cycle checkpoints. The increase in p-ATM (Ser1981) phosphorylation in both U251 and T98G glioblastoma cells suggested its activation after LCS1269 treatment (Figure 6B). Several sites of autophosphorylation (Ser428, Ser435, Thr1989, Ser436, Ser437) during ATR activation have been shown [28]. In our study, p-ATR (Ser428) phosphorylation was markedly increased after LCS1269 treatment in T98G cells, and only slightly increased in U251 cells (Figure 6B).

ATM and ATR are recruited to the DNA damage sites and, after activation, phosphorylate several downstream targets, especially checkpoint kinases Chk1 and Chk2, to promote DNA repair [29]. Another direct downstream target for phosphorylation by ATM and ATR is BRCA1, breast cancer susceptibility protein, a critical component of the checkpoint signaling and DNA repair machinery [30]. In response to DNA damage, BRCA1 is hyperphosphorylated by both ATM and ATR on multiple Ser/Thr residues, and in particular on Ser1524 [31]. The augmentation of p-BRCA1 (Ser1524) in both U251 and T98G was also indicative of DNA damage due to LCS1269 treatment (Figure 6B).

In addition to the exogenous DNA-damaging agents, DNA damage could be induced endogenously by reactive oxygen species (ROS), replication defects, and replication fork collapse. Of particular interest is the damage caused by Top I inhibition. To evaluate the exact mechanism of DNA damage caused by LCS1269, the expression of the proteins participating in the DNA reparation processes was analyzed. Double-strand breaks (DSBs) are repaired by two principal mechanisms: non-homologous end-joining (NHEJ) and homologous recombination (HR) [31,32]. A DNA-binding protein, Rad51 recombinase, forms foci at sites of single-stranded DNA following DNA damage and plays a critical role in HR [33]. The increase in Rad51 expression, as well as significantly inducing Rad51 foci formation in U251 and T98G glioblastoma cells after LCS1269 treatment, indicated the possible participation of the HR mechanism of reparation in DNA damage induced by LCS1269 (Figure 6C,D).

In NHEJ, DSBs are recognized by the Ku protein, a heterodimeric DNA-binding complex made up of two subunits, Ku70 and Ku80 [34,35]. Interestingly, there was a ubiquitous expression of Ku70 and Ku 80 in two analyzed untreated glioblastoma cells, U251 and T98G. LCS1269 treatment had no significant effect on the expression of Ku70 and Ku80 subunits, although Ku70 expression was slightly diminished in U251 cells after LCS1269 treatment (Figure 6C). These data suggest that NHEJ was not activated due to LCS1269 treatment.

**Figure 7:**
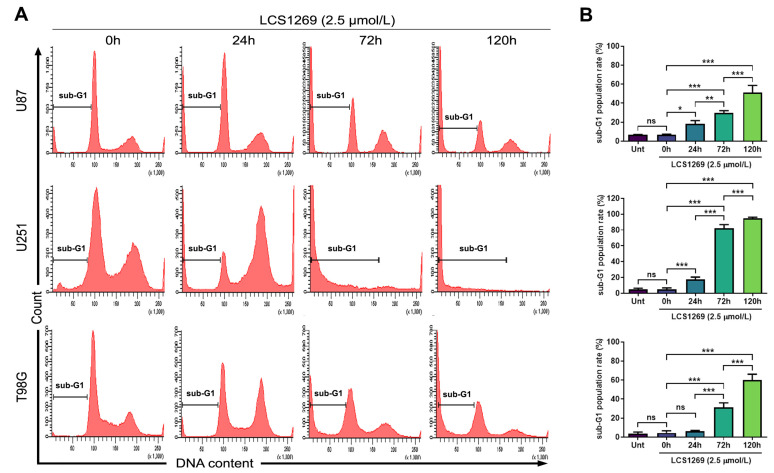
LCS1269 causes the death of glioblastoma cells in a time-dependent manner. (**A**) Representative flow cytometry graphs show the effect of LCS1269 (2.5 µM) exposed for 0, 24, 72, and 120 h on the sub-G1 phase in U87, U251, and T98G cells. (**B**) Quantification of sub-G1 contents in untreated cells and after LCS1269 treatment (2.5 µM) for indicated times. Data presented as mean ± SD; ns—not significant, * *p* < 0.05, ** *p* < 0.01 and *** *p* < 0.001.

We have previously shown that LCS1269 inhibited the cell viability of glioblastoma cells. IC_50_ values for U87, U251, and T98G cells were 14.33 ± 2.52 µM, 0.70 ± 0.08 µM, and 2.83 ± 0.93 µM, respectively [20]. To elaborate on the mechanism of the cell death, we evaluated the content of sub-G1 cells in glioblastoma cell cultures incubated with LCS1269 (2.5 µmol/L) for 24 h, 72 h, and 120 h (Figure 7A). There was no significant difference in the level of sub-G1 contents between vehicle controls (untreated cells) and cells treated for 0 h (Figure 7B). During the first 24 hrs, the number of sub-G1 cells did not exceed 20%. A rapid increase in sub-G1 cells was observed after 72 hrs incubation with LCS1269, reaching a 60% increase in U87 and T98G cells and almost 100% in U251 cells after 120 hrs (Figure 7B).

## 3. Discussion

By using U87 glioblastoma cells xenografted in nude mice, we have previously shown that the LCS1269 treatment considerably restrained tumor growth in vivo [20]. We have also found that LCS1269 triggered G2 cell cycle block associated with Wee1/Myt1 axis activation, the upregulation of transcription factor Forkhead Box M1 (FOXM1), and its target Polo Like Kinase 1 (PLK1) in human glioblastoma cell lines [20]. In this study, we further investigated whether these processes were accelerated due to a genomic instability triggered by direct (as a consequence of DNA biding ability) and/or indirect (as a result of Top I activity modulation) DNA damage effects of LCS1269. It has previously been demonstrated that FOXM1 expression was elevated in response to radiation-induced replicative stress and genomic instability [36,37]. Moreover, several studies reported that the transcriptional target of FOXM1, PLK1, might be frequently upregulated, resulting in chromosomal instability [38,39].

DNA damage is considered to be one of the main triggers of genomic instability. Subsequently, various drugs including indolo [2,3-*a*]pyrrolo [3,4-*c*]carbazole derivatives have been tested to evaluate their effect on DNA stability. To understand the molecular and cellular mechanisms of LCS1269 action, we first investigated its DNA binding ability. Our in silico and ex vivo findings clearly confirmed that LCS1269 is a DNA tropic compound, preferentially interacting with minor grooves of DNA molecules with more affinity to AT-rich regions of DNA. Importantly, the definitive biophysical validation of the LCS1269–DNA interaction was beyond the scope of the present study and will be pursued separately. At the same time, both the ability of LCS1269 to interact with DNA and the DNA affinity constant for LCS1269 were previously found [22].

It is well known that the inhibition of DNA topoisomerases activity, in particular Top I, can also facilitate DNA damage [40,41]. Therefore, several drugs with potential anti-Top I activity are currently being investigated in several clinical trials. Notably, among these are drugs with a molecular structure similar to LCS1269. For example, becatecarin and edotecarin have demonstrated the ability to intercalate into DNA and inhibit Top I activity [42].

To investigate the effect of LCS1269 on Top I enzyme activity, we performed a plasmid DNA relaxation assay. Our results clearly demonstrated that LCS1269 effectively suppressed Top I enzyme activity. Moreover, LCS1269 downregulated the expression of Top I protein in a dose- and time-dependent manner regardless of the proteasomal degradation system. Our docking analysis also suggested that the probability of LCS1269 interacting with Top I, as well as with the Top I/DNA complex, was highly feasible. It is worth noting that the docking results of LCS1269 interaction with Top I/DNA complex predicted the bonds formed by LCS1269 with both the amino acid sequence of Top I and DNA nucleotides, which implies the double anchorage of ligand LCS1269 to the Top I/DNA complex. In summary, the docking analysis indicates a high probability of LCS1269 interaction with the Top I active center, which may lead to the inhibition of Top I activity and, consequently, DNA damage.

Phosphorylated H2AX (p-γH2AX) is a DNA damage sensor which signals the presence of DNA damage and links DNA repair machinery to damaged chromatin [27]. DNA damage activates DNA damage checkpoints and induces cell cycle arrest in G1, S, or at the G2/M phases to promote DNA repair and to prevent the transmission of damaged DNA to daughter cells. To test the effect of LCS1269 on the phosphorylation level of H2AX, we performed Western blot analysis using a specific anti-phospho-γH2AX (Ser139) antibody. Our finding revealed significantly increased phosphorylation of p-γH2AX (Ser139) in response to LCS1269 treatment in a dose-dependent manner in three different glioblastoma cell lines, U87, U251, and T98G. These data convincingly show that LCS1269 contributes to DNA damage in various glioblastoma cell lines.

In response to DNA damage, the autophosphorylation of multiple sites (Ser367, Ser1893, Ser1981, Ser2996, and potentially others) transforms ATM from an inactive dimer into active monomers. S1981 phosphorylation is considered to be the most valuable and widely used marker for ATM activation in cells [43]. ATM is primarily involved in G2 checkpoint arrest [44]. ATR, a downstream target of ATM, is mostly involved in G1 checkpoint arrest and double strand break repair [45]. As expected, LCS1269 treatment upregulated the expression of both ATM and ATR, with a more profound modulation of p-ATM (Ser1981).

Chk1 is specifically expressed at the S to M phase of the cell cycle at both RNA and protein levels. It remains active even in unperturbed cell cycles [46]. In response to DNA damage, Chk1, a specific downstream substrate of ATR, is activated and phosphorylated by ATR. In the absence of ATR kinase activity, Chk1 can be phosphorylated by ATM [47,48]. Both ATM and ATR can phosphorylate Chk1 on Ser317 and Ser345 amino acid residues, inducing Chk1 autophosphorylation on Ser296 [49].

Chk2 is a stable protein that is expressed throughout the cell cycle. It appears to be mostly inactive in the absence of DNA damage and is activated by phosphorylation mainly by ATM in response to double-stranded DNA breaks [50]. Here, Thr68 is the major in vitro Chk2 site phosphorylated by ATM [51]. It has been shown that BRCA1 is recruited to the sites of DNA breaks and co-localized with p-γH2AX foci in response to DNA damage [52]. It is suggested that BRCA1 plays an important role in recruiting kinases responsible for H2AX phosphorylation. BRCA1 regulates the length and the persistence of ssDNA at sites of DNA damage [53]. Similarly, in our experiments, LCS1269 affected p-Chk1 (Ser345) and p-BRCA1 (Ser1524) phosphorylation levels in a dose-dependent manner in both glioblastoma cell lines, U251 and T98G, whereas p-Chk2 (Thr68) phosphorylation was increased only in U251 cells.

Of note, HR in mammalian cells is most active in the S phase of the cell cycle, reaching its maximum in the mid-S phase during active DNA replication and then gradually decreasing as cells progress from the late S to the G2 phase [54]. NHEJ is active during all phases of the cell cycle, predominating in G1 and G2, and is the preferred DSB repair pathway in higher eukaryotes [34]. We revealed that LCS1269-induced DNA damage eventually facilitated preferential Rad51 upregulation (the crucial effector of HR) and Rad51 foci formation, whereas levels of Ku70 and Ku80 subunits of helicase complex involved in NHEJ were marginally changed. At the same time, we found incremental and significant accumulation of sub-G1 content corresponding to dead cells, thus indicating that the Rad51-related mechanism of HR repair is insufficient to repair LCS1269-induced DNA damage.

Altogether, our findings convincingly suggest that the upregulation of key molecules of DNA damage response (DDR) and HR repair detected after LCS1269 treatment reflects a rapid activation of the ATM/Chk1/BRCA1/RAD51 pathway. Meanwhile, an evident insufficiency of the ATM/Chk1/BRCA1/RAD51 pathway as a reparative mechanism eventually culminates in the death of GBM cells.

## 4. Materials and Methods

### 4.1. Cell Lines

The GBM cell lines U87, U251, and T98G were purchased from the American Type Culture Collection (ATCC, Manassas, VA, USA) and cultivated as previously described [20].

### 4.2. Cell Cycle Analysis and Sub-G1 Content Evaluation

The U87, U251, and T98G cells were inoculated at a density of 2 × 10^5^ per well into 6-well plates in complete culture medium, further cultivated, and processed as previously described [17]. Briefly, at the end of incubation time, the attached cells were rinsed twice with PBS, then collected using a 0.25% trypsin-EDTA solution (Paneco, Moscow, Russia) and fixed with 70% ethanol at 4 °C for at least 3 h. After this, the cells were centrifuged at room temperature at 1000 rpm for 5 min, washed once with PBS, and treated with 10 μL of RNAse A (10 mg/mL) for 30 min at 37 °C. The cell pellets were then resuspended in 400 μL of PI solution (0.5 mg/mL in PBS) and incubated for 30 min in the dark. The DNA content was analyzed using a FACSCanto2 flow cytometer (BD Biosciences, Franklin Lakes, NJ, USA), and fluorescence emitted from the PI–DNA complex was estimated at 488 nm using at least 10,000 cells per sample. The sub-G1 content was determined using ModFit LT software, version 3.2 (Verity Software House, Topsham, ME, USA).

### 4.3. Western Blotting

The cells were scraped and lysed using RIPA buffer (ThermoScientific, Waltham, MA, USA). The following procedures were previously described in detail [20]. Briefly, after SDS-PAGE electrophoresis and blotting onto a nitrocellulose membrane, blots were incubated at 4 °C overnight with specific primary antibodies (Supplementary Table S2). After that, the blots were washed with TBS/Tween-20 and incubated at room temperature for 1 h with horseradish peroxidase (HRP)-conjugated secondary antibodies (Jackson ImmunoResearch, West Grove, PA, USA). Membranes were washed with TBST and protein bands were visualized with enhanced chemiluminescence (ECL) substrate (ThermoScientific, Waltham, MA, USA) and imaged by Image Quant LAS4000 (GE HealthCare, Chicago, IL, USA). The β-Actin protein level served as a loading control. ImageJ software, version 2.14.0 (National Institutes of Health, Bethesda, MD, USA), was applied to measure the expression of investigated proteins.

### 4.4. Plasmid DNA Relaxation Assay

Briefly, 10 µL of reaction mixture containing the whole cell extract of MCF7 cells as a source of endogenous Top I was diluted in the relaxation buffer (10 mM Tris-HCl, pH 7.9; 1 mM EDTA; 0.15 M NaCl; 0.1% BSA; 0.1 mM spermidine; 5% glycerol) and incubated with 100 ng supercoiled pBR322 plasmid DNA (SibEnzyme, Novosibirsk, Russia) in the presence of either different LCS1269 concentrations, camptothecin (positive control), or 1% DMSO (solvent control) at 37 °C for 30 min. The reaction was stopped by the addition of proteinase K (Syntol, Moscow, Russia) to a final concentration of 100 µg/mL and subsequently heated at 60 °C for 30 min. Thereafter, the samples were resolved on 1% agarose gel with 1× TAE buffer (40 mM tris-base, 1 mM EDTA, 20 mM acetic acid) and photographed under UV light after staining in ethidium bromide solution (0.5 μg/mL).

### 4.5. DNA Competition Studies

DNA binding properties in the presence of different LCS1269 concentrations were assessed by spectrofluorometry with a SpectraMax M5e microplate reader (Molecular Devices, San Jose, CA, USA). Ethidium bromide (EtBr) and Hoechst 33,259 (Sigma-Aldrich, Saint Louis, MO, USA) were used as common intercalator and minor groove binder, respectively. The double-stranded Salmon sperm (SS) DNA (Sigma-Aldrich, Saint Louis, MO, USA) concentration was determined by absorption spectroscopy, assuming the molar extinction coefficient at 260 nm as 6600 (mol/L)^−1^ cm^–1^. The DNA solution displayed a ratio of UV absorbance at 260 nm and 280 nm greater than 1.9, suggesting that the DNA was sufficiently free of protein impurities (Supplementary Figure S4). In both fluorescence displacement experiments, LCS1269 was added at increasing concentrations to the mixture containing 50 μM SS-DNA and EtBr (1 µM) or Hoechst 33,259 (10 µM) in 10 mM Tris-HCl (pH 7.4) buffer. The mixture was further incubated for 15 min and the resulting fluorescence detected at λ_ex_ at 300 nm and λ_em_ at 600 nm or at λ_ex_ of 350 nm and λ_em_ of 450 nm for EtBr and Hoechst 33259, respectively.

### 4.6. pBR322 Gel Migration Assay

The mixture of LCS1269 in the indicated concentration, irinotecan (Sigma-Aldrich, Saint Louis, MO, USA) as a negative control (100 µM), or DB (7) as a positive control (10 µM) with 100 ng pBR322 plasmid DNA (SibEnzyme, Novosibirsk, Russia) in Tris-EDTA buffer (pH 7.4) was incubated at room temperature for 1 h. The samples were electrophoresed in 1× TAE buffer (40 mM tris-base, 1 mM EDTA, 20 mM acetic acid) by 1% agarose gel. Then, the samples were stained with an aqueous solution of ethidium bromide (0.5 μg/mL) and visualized using UV light.

### 4.7. Rad51 Foci Formation

U251 cells cultured on coverslips were treated with (2.5 µM) or without LCS1269 for 24 h. Fixation, permeabilization, and incubation conditions with primary and secondary antibodies as well as immunofluorescent visualization procedures were previously described [55]. To counterstain the nuclei of cells and F-actin filaments, DAPI (Sigma-Aldrich, Saint Louis, MO, USA) and Phalloidin Alexa Fluor^™^ 594 (ThermoScientific, Waltham, MA, USA) were used, respectively. The quantification of nuclei containing ≥5 Rad51 foci was performed manually in at least 10 random microscopic fields.

### 4.8. Molecular Docking

The structures of Top I in covalent complex with 22-pair DNA duplex (Protein Data Bank (PDB ID: 1A31)) and DNA duplexes (PDB ID: 1BNA, PDB ID: 1D29, and PDB ID: 3EYO) as putative targets of the LCS1269 ligand were taken from the open database PDB (https://www.rcsb.org/, accessed on 15 March 2025) and analyzed as previously described [20].

### 4.9. Statistical Analysis

All data were expressed as the mean ± SD and analyzed with GraphPad Prism software, version 5.02 (GraphPad Software, San Diego, CA, USA). Each experiment was independently repeated at least three times. Statistical comparisons were analyzed by one-way analysis of variance (ANOVA) followed by a Newman–Keuls multiple comparison post hoc test where necessary. Statistical significance was defined as * *p* < 0.05, ** *p* < 0.01, *** *p* < 0.001.

## 5. Conclusions

In the present study, we have demonstrated that LCS1269 may interact with DNA as a minor groove binder rather than an intercalator. Our in silico and ex vivo approaches confirmed that LCS1269 could preferentially target both AT-rich sequences and the minor groove of DNA. Moreover, we showed that LCS1269 was able to affect Top I activity through both the direct inhibition of its enzyme activity and the downregulation of the Top I protein level. LCS1269 also induced DNA damage in GBM cells followed by DDR activation. Meanwhile, DDR activation facilitated the induction of the homologous recombination mechanism, and both Rad51 recombinase upregulation and significantly induced Rad51 foci formation in GBM cells. However, the initiation of Rad51-associated homologous recombination repair was insufficient. All GBM cells gradually died in a time-dependent manner after LCS1269 treatment. Hence, our results clearly suggest that both DNA molecule and Top I are the potential targets for the anti-glioblastoma activity of LCS1269.

## Data Availability

The original contributions presented in this study are included in the article/Supplementary Material. Further inquiries can be directed to the corresponding author.

## Supplementary Materials

The following supporting information can be downloaded at https://www.mdpi.com/article/10.3390/ijms26136014/s1.
