# Supplementary material for: Targeting Topoisomerase I and DNA with LCS1269 Drives Glioblastoma Cell Death Despite ATM/Chk1/BRCA1/RAD51 Signaling Pathway Activation
Source: Int J Mol Sci. 2025 Jun 23;26(13):6014. doi: 10.3390/ijms26136014 (PMC12249491; doi:10.3390/ijms26136014)
Supplement: Supplementary file 1 [file ijms-26-06014-s001.zip › ijms-3628815-supplementary.pdf]

Targeting topoisomerase I and DNA with LCS1269 drives glioblastoma cell death despite ATM/Chk1/BRCA1/RAD51 signaling pathway activation

Nikolay Kalitin [1], Ekaterina Savchenko [2], Nadezhda Samoylenkova [2], Natalia Koroleva [3], Anna Lushnikova [3], Aida Karamysheva [1] and Galina Pavlova [2, 4]

[1] Laboratory of Tumor Cell Genetics, N.N. Blokhin National Medical Research Center of Oncology, Moscow, Russia

[2] Laboratory of Molecular and Cellular Neurogenetics, N.N. Burdenko National Medical Research Center of Neurosurgery, Moscow, Russia

[3] Laboratory of Oncogenomics, N.N. Blokhin National Medical Research Center of Oncology, Moscow, Russia

[4]Laboratory of Neurogenetics and Developmental Genetics, Institute of Higher Nervous Activity and Neurophysiology of RAS, Moscow, Russia

Corresponding author:

Nikolay Kalitin, PhD, N.N. Blokhin National Medical Research Center of Oncology, 24 Kashirskoe Shosse, 115478 Moscow, Russia. E-mail: f.oskolov@mail.ru

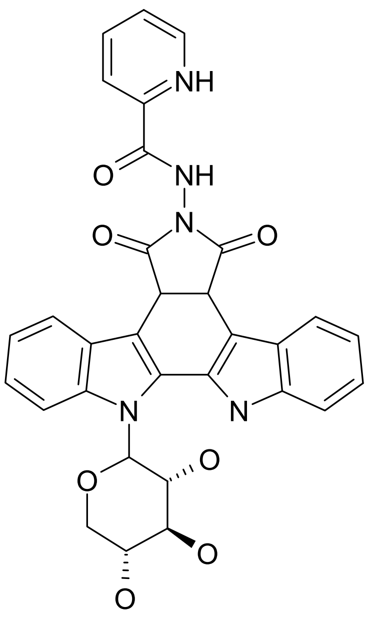

Figure S1. Molecular structure of N-[5,7-dioxo-12-(*β*-*D*-xylopyranosyl)-5,7,12,13-tetrahydro-6H-indolo [2,3-*a*]pyrrolo [3,4-*c*]carbazole-6-yl]pyridine-2-carboxamide (LCS1269)

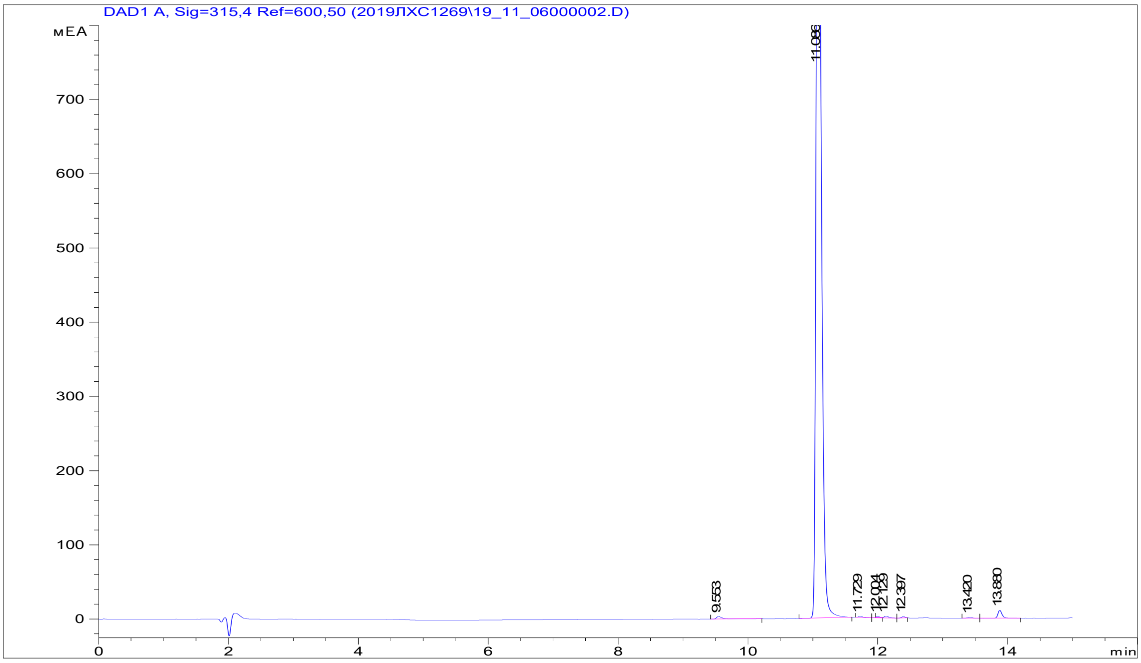

Figure S2. HPLC analysis of LCS1269. LCS1269 purity was 98 – 99%. The gradient elution at 40°C was carried out. The gradient started at water/acetonitrile (9:1) + trifluoroacetic acid 0.1% (mobile phase A) and ended at water/acetonitrile (0.5:9.5) + trifluoroacetic acid 0.1% (mobile phase B) after 15 min. The retention time was 10.9 ± 0.2 min, λ 315 nm.

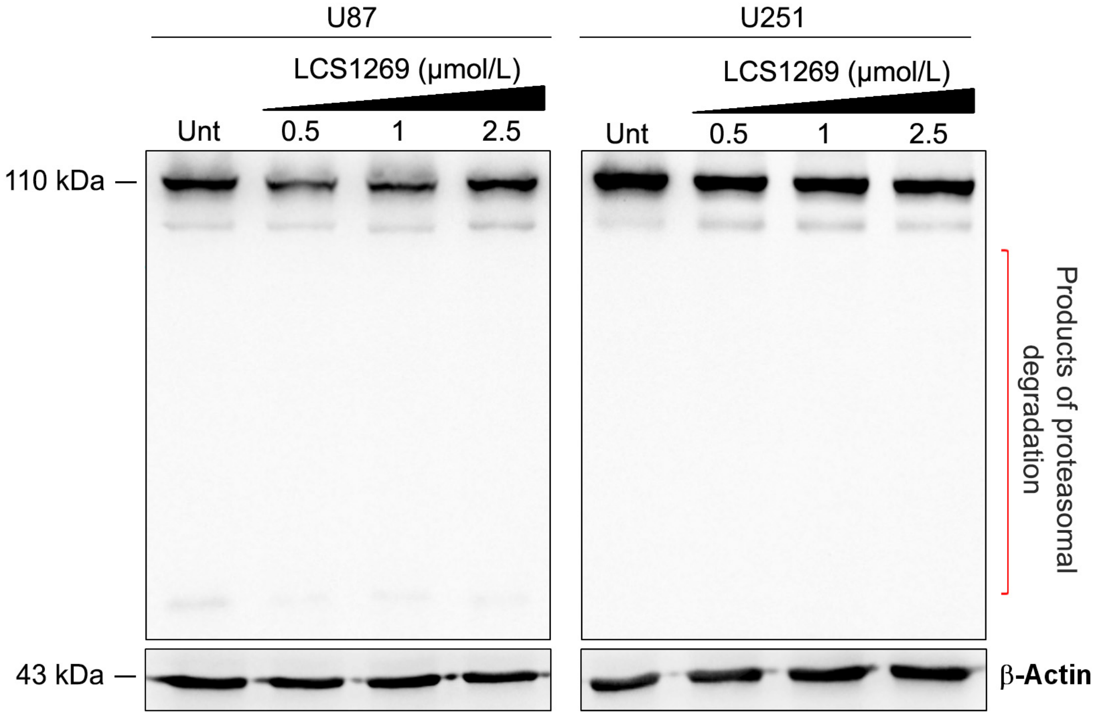

Figure S3. Impact of LCS1269 treatment on the induction of proteosomal-dependent degradation of Top I. Western blot analysis of Topoisomerase I protein levels from U87 and U251 cells without treatment (Unt) or treated with LCS1269 (0.5, 1, and 2.5 μM) for 24h. β-Actin served as a loading control.

Table S1. Glide scores for putative interactions simulated between LCS1269 and DNA duplexes

**DNA duplex[a]**	**Score**
1BNA	–6.39
1D29	–6.64
3 EYO	–8.35

[a]Protein Data Base (PDB) ID number

Table S2. Primary antibodies used in Western blot analyses

Antibody name	Manufacturer	Catalogue number	Dilution rate
p-Histone H2A.X (Ser139) (20E3) Rabbit mAb	Cell Signaling Technology	9718	1:1000
p-Chk1 (Ser345) (133D3) Rabbit mAb	Cell Signaling Technology	2348	1:1000
p-Chk2 (Thr68) (C13C1) Rabbit mAb	Cell Signaling Technology	2197	1:1000
p-ATM (Ser1981) (D25E5) Rabbit mAb	Cell Signaling Technology	13050	1:1000
p-ATR (Ser428) Antibody	Cell Signaling Technology	2853	1:1000
p-BRCA1 (Ser1524) Antibody	Cell Signaling Technology	9009	1:1000
Rad51 (D4B10) Rabbit mAb	Cell Signaling Technology	8875	1:1000
Ku70 (D10A7) Rabbit mAb	Cell Signaling Technology	4588	1:1000
Ku80 Antibody	Cell Signaling Technology	2753	1:1000
TOP1 Polyclonal Antibody	ThermoScientific (Invitrogen)	PA5-82658	1:1000
beta-Actin (C4) Mouse mAb HRP	Santa Cruz Biotechnology	sc-47778 HRP	1:500

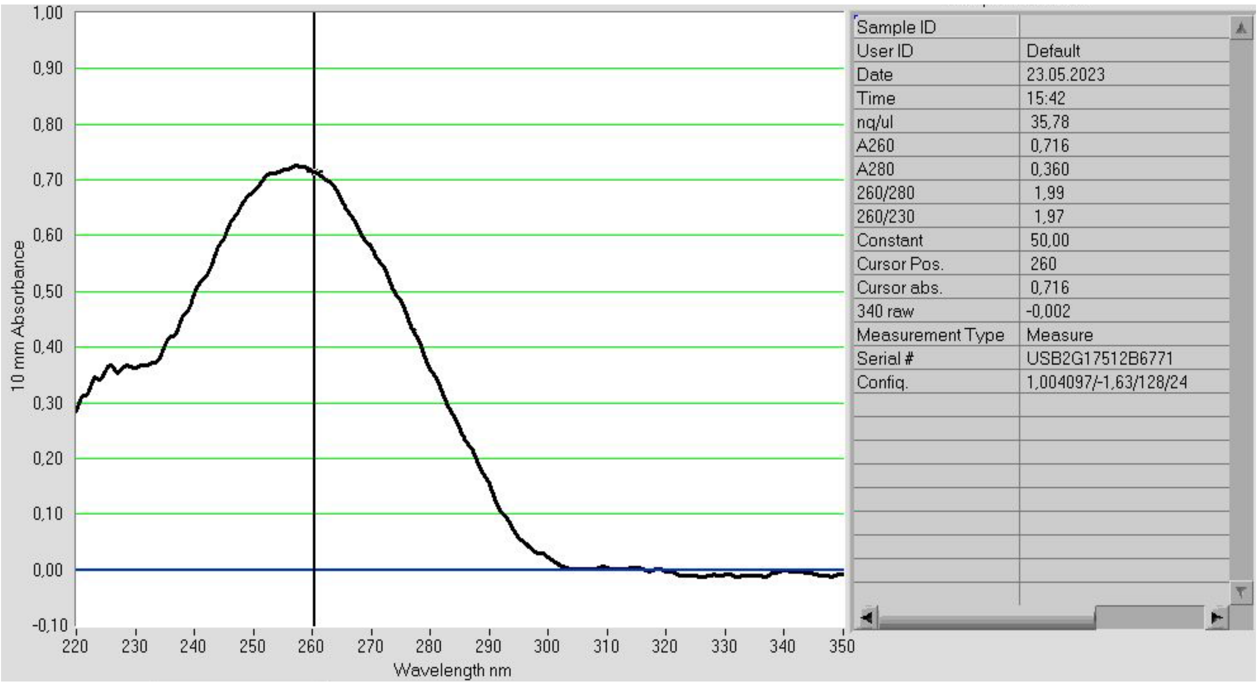

Figure S4. UV absorbance spectra of *Salmon sperm* DNA measured by NanoDrop 1000 spectrophotometer

Supplementary Note 1

Earlier, Zenkov et al. [1] using FID assay [2] investigated affinity of LCS1269 to DNA oligonucleotide duplex. They defined that the binding constant of LCS1269 with DNA equals $(2.52 \pm 0.4) \times 10^5$ M^{-1} [1].

Moreover, Zenkov et al. [1] also analyzed the mechanism of interaction between LCS1269 and DNA by means of circular dichroism in cholesteric liquid crystals (CD in CLC) [3]. The authors confirm that CD spectrum in the presence of LCS1269 demonstrates a decrease of the DNA peak [1]. It is the evidence of LCS1269 binding to DNA [1]. However, the low peak of LCS1269 did not enable to determine unambiguously the binding mode with DNA for this compound.

Supplementary references

1. Zenkov, R.; Vlasova, O.; Maksimova, V.; Fetisov, T.; Karpechenko, N.; Ektova, L.; Eremina, V.; Popova, V.; Usalka, O.; Lesovaya, E.; et al. Molecular Mechanisms of Anticancer Activity of N-Glycosides of Indolocarbazoles LCS-1208 and LCS-1269. *Molecules.* **2021**, *26*, 7329.
2. Monchaud, D.; Allain, C.; Teulade-Fichou, MP. Development of a fluorescent intercalator displacement assay (G4-FID) for establishing quadruplex-DNA affinity and selectivity of putative ligands. *Bioorg Med Chem Lett*. **2006**, *16* 4842–4845.
3. Yevdokimov, Y.; Skuridin, S.; Nechipurenko, Y.; Zakharov, M.; Salyanov, V.; Kurnosov, A.; Kuznetsov, V.; Nikiforov, V. Nanoconstructions based on double-stranded nucleic acids. *Int J Biol Macromol*. **2005**, *36*, 103–115.
